# A Retrospective Analysis of Chest Radiographic Patterns in Patients With COVID-19

**DOI:** 10.7759/cureus.78942

**Published:** 2025-02-13

**Authors:** Narender Singh, Saroj Kumar Pati, Narendra Kuber Bodhey, Sajal De, Ajoy K Behera

**Affiliations:** 1 Radiodiagnosis, All India Institute of Medical Sciences, Raipur, IND; 2 Pulmonary Critical Care and Sleep Medicine, All India Institute of Medical Sciences, Raipur, IND; 3 Pulmonary Medicine and Tuberculosis, All India Institute of Medical Sciences, Raipur, IND

**Keywords:** chest radiography, consolidation, covid-19 pneumonia, ground glass opacities, modified rales

## Abstract

Background: Chest radiography is often the most utilized and primary investigation for patients with COVID-19 pneumonia, however, only limited studies are available evaluating its essence. Therefore, we retrospectively analyzed various chest radiographic patterns in patients with COVID-19 and correlated the radiographic severity index with clinical severity and laboratory parameters.

Methods: In this retrospective study, radiographs of 512 COVID-19 patients diagnosed with pneumonia were assessed, out of which 289 patients satisfying inclusion and exclusion criteria were recruited for the study. The spectrum of radiographic findings was compared with the contemporary clinical and laboratory records.

Results: Ground glass opacities (GGOs; 250/289, 86.5%) and consolidations (166/289, 57.4%) were the most common findings seen in radiographs, with the most common distributions being “basal and peripheral” (92/289, 31.9%), followed by “non-specific pattern” (73/289, 25.3%), “basal” (60/289, 20.8%), and “peripheral” (48/289, 16.6%) patterns. A statistically significant association was seen between the clinical and radiographic severity scores and in-hospital mortality and radiographic severity scores. Also, a statistically significant association was seen between the radiographic severity score and various laboratory parameters (i.e., white blood cell (WBC) count, lactate dehydrogenase (LDH), C-reactive protein (CRP), and erythrocyte sedimentation rate (ESR)).

Conclusion: With this study, we concluded that specific patterns of lung involvement were seen in patients with COVID-19 and that radiographic severity scores correlated well with the clinical severity and laboratory parameters. Hence, in our opinion, chest radiography could be a useful tool for stratifying disease severity and differentiating between severe and non-severe COVID-19 pneumonia.

## Introduction

COVID-19 is caused by severe acute respiratory syndrome coronavirus 2 (SARS-CoV-2), belonging to the coronavirus family, with its first case reported in China on 31^st ^December 2019. The disease spread rapidly and was declared a pandemic by the WHO on 11^th^ March 2020 [1]. India reported its first case in January 2020, and after that, the world was hit by three consecutive waves in the next two years. There have been more than 249 million total reported COVID-19 cases globally with 4.9 million deaths [2,3].

SARS-CoV-2 has an affinity for angiotensin‐converting enzyme 2 (ACE-2) receptors present in type II alveolar cells. After entering the alveolar cells, it results in diffuse alveolar damage with exudates filling the alveolar space [4]. Radiologically, it is seen as ground glass opacities (GGOs) on both radiographs and computed tomography (CT) scan images in the early course of the disease. As the disease progresses, the alveolar cells die, and exudates may result in consolidation. These consolidations generally do not incite pleural effusion or cavitations as seen in bacterial infections [5].

For diagnosis of COVID-19, real-time polymerase chain reaction (RT-PCR) assay remains the gold standard investigation. Since there is a paucity of RT-PCR availability, radiological imaging like CT scans and chest radiography have been extensively used for the diagnosis and management of COVID-19 disease [6]. Moreover, chest radiography was found to have fair to good interobserver variability while assessing COVID-19 [7-9].

A CT scan is an excellent modality for the evaluation of pulmonary parenchymal changes. However, multiple limiting factors like limited availability in the rural and peripheral parts of developing countries including India, difficulties related to imaging of critically ill patients, high radiation dose to the patients, high cost, and longer time taken in CT room decontamination; limit its widespread use. Hence, chest radiography remains the most cost-effective modality for the management of COVID-19 pneumonia [10].

Multiple studies were published highlighting the importance of chest radiography for the evaluation of disease. There have been encouraging results in these studies, particularly in terms of specific patterns of lung involvement and severity assessment of the disease. Most of these studies focus mainly on the Brixia and Radiographic Assessment of Lung Edema (RALE) scoring system [7-13]. Here, we conducted this retrospective study intending to interrogate the chest radiographic patterns and correlate the radiographic severity with clinical and lab parameters in COVID-19-affected patients.

## Materials and methods

Study population

This retrospective study was conducted at All India Institute of Medical Sciences, a tertiary care center in Raipur, Central India, following approval from the Institutional Ethics Committee and a waiver of informed consent (letter No.:2049/IEC-AIIMSRPR/2021, dated 30.11.2021). Data were retrieved from the institute's medical record database from March 2021 to June 2021. A total of 512 cases were consecutively selected from the archives of patients admitted to our hospital with the diagnosis of COVID-19 pneumonia during this period.

Out of these 512 cases, 289 patients satisfying the inclusion and exclusion criteria were recruited for the study. The inclusion criteria were patients admitted with microbiologically confirmed (by either RT-PCR test or rapid antigen test for COVID-19 infection) cases and underwent chest radiography. The patients were excluded if clinical records and laboratory parameters were not available, age <18 years and available radiographs were grossly suboptimal for reading. Contemporary clinical and laboratory records of the selected patients were retrieved from the medical records and evaluated.

Equipment used

All the radiographs were acquired in supine anteroposterior (AP) projection, using a portable X-ray unit (SkanMobile, SkanRay Technologies Ltd., Bangalore, India) analogue cassette-based radiographic acquisition system, which was coupled with a computed radiography (CR) cassette system. Images were stored in Digital Imaging and Communications in Medicine (DICOM) format.

Radiograph evaluation and scoring

Archived images were retrieved in DICOM format and interpreted by two investigators with more than 10 years and 25 years of experience who were blinded to clinical and laboratory findings. The results were compared, and any disagreements were resolved through consensus. A detailed analysis of the radiographic features and predominant patterns was observed on each radiograph. Radiographic features including consolidation, GGOs, pulmonary nodules, and reticulo-nodular opacities were evaluated according to the Fleischner Society glossary of terms [11]. Further, the radiographs were given severity scores according to the modified RALE scoring system (Table 1) [14]. The maximum severity score of the radiograph during a patient's hospital stay was used for assessment, without considering whether multiple radiographs were taken at different times or the status of the patient at each specific time point. Calculated radiographic scores were correlated with the clinical severity grades and in-hospital patient demise, if any. Correlation was also assessed between the radiographic severity stage and laboratory parameters.

**Table 1:** Modified RALE score for staging of the disease on radiographs The table is adapted from the article by Wong et al [14]. RALE: Radiographic Assessment of Lung Edema

Percentage of lung field involves	Score provided	Final score (left+right)	Disease staging
0%	0	0-2	Mild
<25%	1	3-5	Moderate
25%-50%	2	6-8	Severe
50%-75%	3		
>75%	4		

Statistical analysis

Data were analyzed using IBM SPSS Statistics software for Windows, version 21 (IBM Corp., Armonk, NY). The statistical analysis was comprised of calculating means and proportions. The Kolmogorov-Smirnov test was used to assess the normality of the data. For non-skewed data, the ANOVA test; for skewed data, the Kruskal-Wallis test; and for categorical data, the chi-square test were used. A p-value ≤ 0.05 was considered statistically significant.

## Results

Demographic characteristics

Out of 289 confirmed patients with COVID-19 in our study, 80 (30.4%) were females and 201 (69.6%) were males. The mean age of the study population was 48.6 ± 14.4 years, with minimum and maximum ages being 20 and 85 years, respectively. The most common comorbidity associated with our study population was either diabetes mellitus only or in combination with hypertension. Patients were categorized into mild, moderate, and severe grades based on WHO severity classification (Table 2) [3]. Out of 289 cases studied, 68 patients died during in-hospital stay.

**Table 2:** Clinical profile of the study population *In our study no case was reported in the age group of 18-20 years during the subject recruitment period.

Population characteristics		Study population n (percentage)
Age	20-30 years*	34 (11.8%)
	31-40 years	63 (21.8%)
	41-50 years	60 (20.8%)
	51-60 years	63 (21.8%)
	61-70 years	49 (17.0%)
	>70 years	20 (6.8%)
Sex	Male	201 (69.6%)
	Female	88 (30.4%)
Comorbidity	Diabetes mellitus	40 (13.8%)
	Both diabetes mellitus and hypertension	40 (13.8%)
	Hypertension	28 (9.7%)
	Chronic kidney disease	5 (1.7%)
	Coronary artery disease	4 (1.4%)
	Tuberculosis	3 (1%)
	Antenatal cases	3 (1%)
Clinical severity of disease (WHO) [12]	Mild	15 (5.2%)
	Moderate	86 (29.8%)
	Severe	188 (65%)

Radiographic characteristics

Out of a total of 289 patients, 12 (4.1%) had normal radiographs, while 251 (87%) had bilateral and 26 (8.9%) had unilateral lung involvement. The most common radioopacity in our study was GGOs in 250 (86.5%), followed by consolidations in 166 (57.4%) and nodules in 35 (12.1%) cases. Other findings were cavitations in four (1.7%) and pleural effusion in three (1.0%) cases. The most common pattern of distribution in our study was “peripheral and basal predominance” in 92 (31.9%) followed by “non-specific pattern” in 73 (25.3%) cases. Other common patterns were “basal predominance” in 60 (20.8%) and “peripheral predominance” in 48 (16.6%) cases. “Apical predominance” and “central predominance” were the least common patterns observed in two patients in each (0.7%).

**Figure 1:**
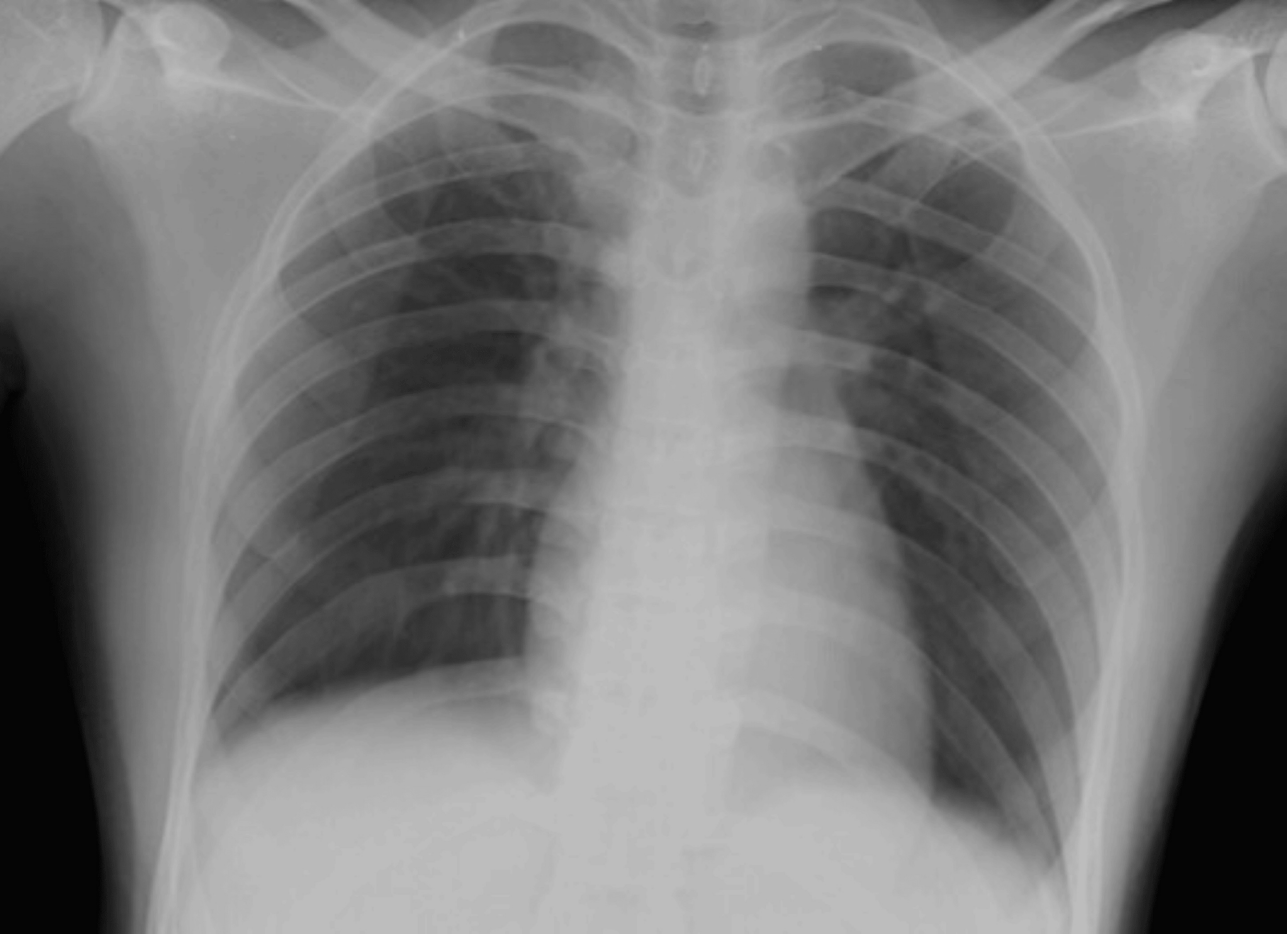
The supine chest radiograph (anteroposterior (AP) view) of a 23-year-old female patient showed no obvious radiopacities in both lung fields. Radiographic disease score was 0.

**Figure 2:**
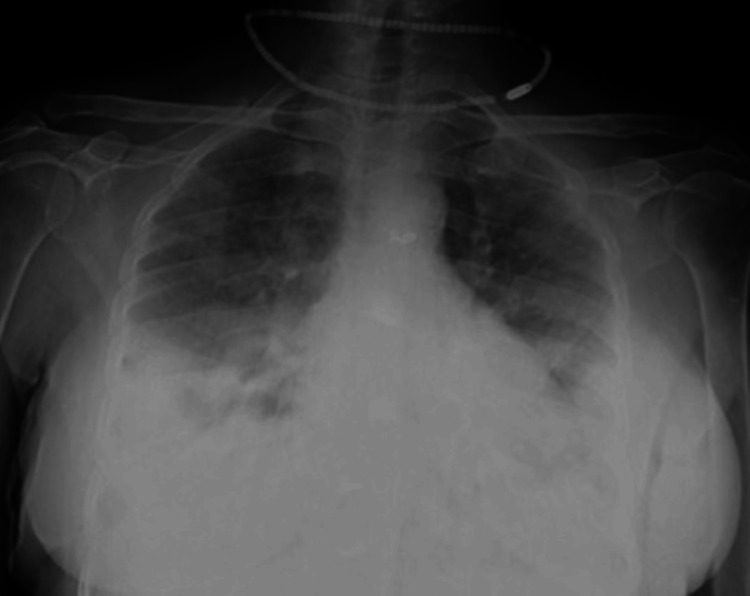
The supine chest radiograph (anteroposterior (AP) view) of a 64-year-old female patient revealed multiple confluent fluffy radiopacities in the bilateral lower lung zones, with peripheral predominance. Ill-defined ground glass opacities were seen in the medial parts of bilateral lower lung zones and bilateral mid-zones. Radiographic disease score=4 (moderate grade) as involvement was noted in both sides was 25% to 50%.

**Figure 3:**
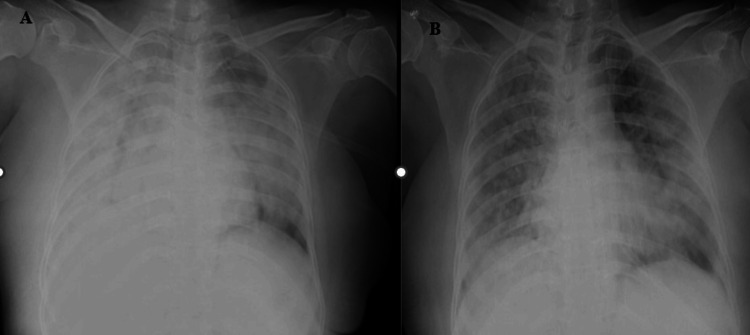
Figure 3A: The supine chest radiographs (anteroposterior (AP) view) of a 53-year-old male patient revealed diffuse ill-defined heterogenous opacities with few linear lucencies likely air bronchogram suggestive of consolidations in bilateral lung parenchyma involving > 75%. Radiographic disease severity score was eight (severe grade). Figure 3B: Follow-up AP supine chest radiographs of the same patient after seven days revealed areas of consolidations and ill-defined nodules in bilateral lung parenchyma involving > 75%.

**Figure 4:**
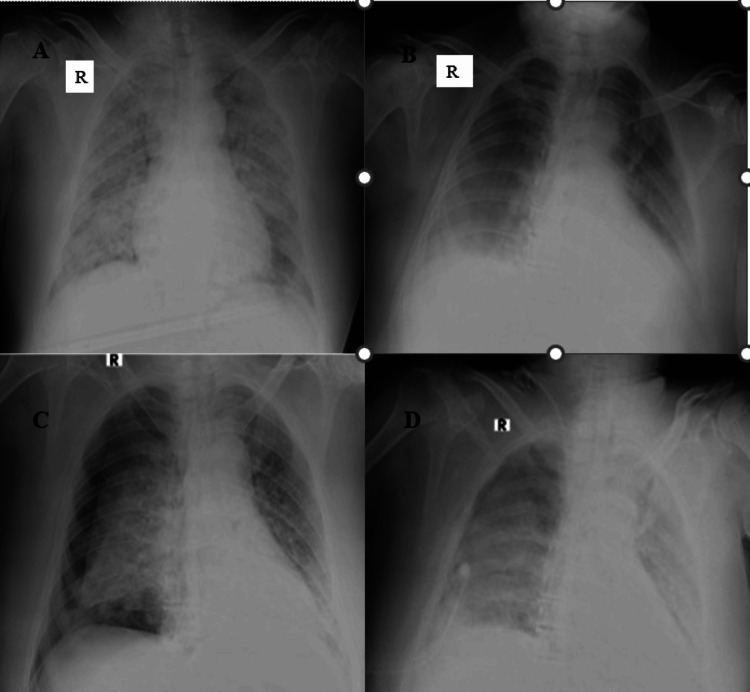
Figure 4A: Chest supine radiograph (anteroposterior (AP) view) of a 64-year-old male patient showed multiple nodular opacities, ill-defined ground glass opacities, and patchy consolidation areas in bilateral lung parenchyma involving > 75% of parenchyma. Radiographic disease severity score was eight (severe grade). Figure 4B: Follow-up chest AP supine radiograph of the same patient on day 10 with mechanical ventilation revealed diffuse areas of GGOs in the bilateral mid and lower lung zones with peripheral and basal high-density areas, suggestive of consolidation. The lung involvement was 25% to 50% bilaterally, and score four represented radiographic improvement. Figure 4C: Follow-up chest AP supine radiograph of the same patient on day 12 showed the development of pneumothorax on the right side (possibly barotrauma-related). Figure 4D: Follow-up chest AP supine radiograph of the same patient on day 14 revealed re-expansion of the right lung with minimal residual pneumothorax. Diffuse ground glass opacities were seen in the entire right lung field, and diffuse areas of consolidation were seen in the entire left lung field.

The severity of COVID-19 was evaluated as per the modified RALE scoring system. Sixty patients were in the “mild” category, while 112 patients belonged to the “moderate” category, and 117 to the “severe” category. A few examples are shown in Figures 1-4.

Zonal distributions of common radiopacities (GGOs and consolidations) with respect to radiographic severity are tabulated in Table 3.

**Table 3:** Zonal distribution of radiopacities (ground glass opacity and consolidation) in radiographs based on the severity score

Radiographic severity	Involved zone		
	Upper zone	Mid zone	Lower zone
Mild (n=60)	2 (3.3%)	20 (33.3%)	45 (75%)
Moderate (n=112)	37(33.0%)	95 (84.8%)	110 (98.2%)
Severe (n=117)	88(75.2%)	115 (98.2%)	113 (96.6%)

Laboratory characteristics

Laboratory parameters like white blood cell (WBC) counts, lactate dehydrogenase (LDH), C-reactive protein (CRP), and erythrocyte sedimentation rate (ESR) were seen to have significantly increased with the radiographic severity of the disease and were found to be statistically significant. On the other hand, D-dimer neither showed such a trend nor was statistically significant (Table 4).

**Table 4:** Laboratory parameters in comparison with radiographic severity *Kuskal-Wallis test; SD: standard deviation

Laboratory parameters (study population)	Mild (mean±SD)	Moderate (mean±SD)	Severe (mean±SD)	Correlation with radiographic severity stage p-value^*^
White blood cell (WBC) (x10^3^cells/μL)	16.9±51.4	11.1±6.2	25.4±99.6	0.01
Lactate dehydrogenase ( LDH) (U/L)	306.9±170.9	501.2±243.5	612.7±300.0	<0.01
C-reactive protein ( CRP) (mg/dL)	45.8±48.8	61.8±49.3	81.9±62.0	<0.01
Erythrocyte sedimentation rate ( ESR) (mm at 1 hr)	46.8±37.4	61.7±39.4	75.1±42.5	<0.01
D-dimer (µg/mL)	1.9±5.8	5.5±31.9	5.9±21.0	0.56

Radiographic and clinical severity association assessment

The distribution of patients in each disease severity category was found to be statistically significant while comparing the radiographic and WHO severity grading (Table 5).

**Table 5:** Comparison of radiographic severity grading and WHO clinical severity grading *Chi-square test

Disease severity	WHO severity grading frequency (percentage)	Radiographic severity grading frequency (percentage)	P-value^*^
Mild	15 (5.2)	60 (20.8)	<0.001
Moderate	86 (29.8)	112 (38.8)	0.023
Severe	188 (65.0)	117 (40.5)	<0.001
Total	289 (100.0)	289 (100.0)	-

Although a significant statistical association was seen between the radiographic and WHO disease severity grades with a p-value of <0.001, a concordance of 56% was noted in our study (Table 6).

**Table 6:** Radiographic and clinical severity association assessment *Z proportion test

WHO severity	Severity on radiograph			Total	p-value^*^
	Mild (0-2)	Moderate (3-5)	Severe (6-8)		
Mild	14	1	0	15	<0.001
Moderate	35	42	9	86	
Severe	11	69	108	188	
Total	60	112	117	289	

The odds ratio for patient demise was 5.8 times higher in the radiographic severe category as compared to the mild category and was found to be statistically significant. It was 1.5 times higher in radiographic moderate disease but not statistically significant (Table 7).

**Table 7:** Evaluation of the association of radiographic severity score with clinical outcome *Chi-square test

Grading	Severity	Death	Odds ratio	p-value^*^
Radiographic severity	Mild	6 (10.0%)	1 (Ref)	-
	Moderate	16 (14.3%)	1.50 (0.55-4.06)	0.42
	Severe	46 (39.3%)	5.83 (2.32-14.65)	<0.01

## Discussion

This retrospective study revealed that GGOs, consolidation, and nodules were the common opacities observed predominantly in peripheral and basal locations involving both lungs. The severity of the disease on the chest radiograph was found to be statistically in correlation with the clinical severity.

The mean age in our study population was found to be in the middle-aged adult group similar to previous studies [12,13]. This might be due to the more prevalent comorbidities like diabetes and hypertension in this age group, which play an important role in disease severity, as noted by previous authors [2, 4, 12].

COVID-19 infection has a predilection for bilateral lung parenchyma, frequently involving lower lung zones as noted in our study as well as in previous literature [5,8,10,11]. Similar to other viral infections, multifocal GGOs and consolidations were observed to be the most frequent radiographic findings in our study, which aligns with the results of previous studies [14-16]. COVID-19 was believed to induce lymphocytic endotheliitis, which results in opacification of alveolar cells [4,15]. This, in the early stage, appears as irregular opacities in the form of GGOs and later as consolidation.

In concurrence with previous studies, we observed that “peripheral and basal” distribution was the most common finding in our study [10,11,14,15]. Other inflammatory pathologies like organizing pneumonia and other atypical pneumonia, including severe acute respiratory syndrome (SARS) and Middle East respiratory syndrome (MERS), simulate COVID-19 on imaging [17]. Therefore, the mere presence of typical radiographic findings was not sufficient enough to diagnose the disease but significant enough to raise suspicion in an appropriate clinical setting.

Pleural effusion, an uncommon complication of COVID-19, has been observed in 2% to 18% of cases [13, 14, 18]. An overall incidence of pleural effusion from meta-analyses was found to be 7.3% by Chong et al. and 5.8% by Bao et al. [19, 20]. We found <1% of cases with pleural effusion in our study, close to a retrospective study stated by Wong et al. [14]. This variation in reported incidence may reflect differences in study populations, methodologies, or diagnostic criteria utilized. We reported a much lower incidence rate, which might be due to the use of AP and supine radiographic techniques. Initially, it was thought to be due to co-existing bacterial infection, but recent studies revealed it might be due to leaky microvasculature or due to direct invasion [18]. Although pleural effusion was more often seen in critically ill patients, the mere presence of pleural effusion could not be related to poor outcomes [19,20]. However, we were unable to establish such a causal relationship due to the rare occurrence in our study population.

Similar to a previous study, we also observed cavitation, a rare manifestation in COVID-19 pneumonia and mostly due to co-infections like *Mycobacterium tuberculosis*, as noted in our case [21].

Barotrauma is a well-known complication seen in patients with COVID-19 more often associated with assisted mechanical ventilation. Loss of lung compliance and hypoxic vasoconstriction regulatory mechanisms possibly cause over-expansion and later rupture of alveoli in the consolidated part of the lungs. This results in pneumothorax, pneumomediastinum, pneumopericardium, pulmonary interstitial emphysema, or subcutaneous emphysema depending on the location of the leaked air. Its incidence rate was found to be as high as 24% in previous studies [15,22-25]. We reported barotrauma in 2.7% of patients (Figure 4C), similar to Kohli et al. [15].

In our study, we found significant correlations between chest radiographic severity grades and various lab parameters, including WBC counts, LDH, CRP, and ESR, with the exception of D-dimer (Table 4). These markers are considered non-specific, as they are involved in the inflammatory response in various multisystem inflammatory syndromes, including COVID-19 pneumonia. Nevertheless, our findings, along with those from previous studies, suggest that the severity of the disease is associated with elevated levels of these inflammatory markers [26,27]. In contrast, the poor correlation of D-dimer in our study could be attributed to various chronic factors like previous medical conditions, old age, and prolonged bed rest for any other cause [28,29]. Moreover, we observed an unusually low WBC count in the moderate chest radiographic disease severity group, which may have occurred by chance. However, consistent with previous studies, we found that severe disease was associated with leukocytosis [26,27].

While a statistically significant association between the radiographic severity score and the WHO clinical severity grading was observed, a notable degree of discordance (44%) was also found. This indicates that while radiographic scoring cannot fully replace clinical grading, it may still serve as a valuable adjunct in stratifying disease severity, offering additional insights into disease progression and aiding in treatment planning.

Radiographic findings of moderate severity showed a poor association with clinical outcomes (death or recovery), which might be due to an insufficient sample size in our study population. In contrast, severe radiographic disease was found to have a good association with clinical outcomes, suggesting that chest radiographs can be useful in predicting severe COVID-19 cases as opposed to non-severe ones. Yong et al. in their study compared all three radiographic scoring systems in ICU patients and showed near-similar findings. The differences may be attributed to the inclusion of only ICU cases in their study population [30].

## Conclusions

This retrospective study revealed that the modified RALE scoring system seems to be a useful tool for stratifying disease severity and differentiating between severe and non-severe COVID-19 pneumonia based on chest radiography. It's interesting that the radiographic changes observed could correspond with laboratory results, which might offer a better understanding of disease severity. Therefore, in our opinion, semiquantitative methods, which don't require complex equipment or highly specialized training, could indeed be a valuable alternative for assessing conditions like COVID-19. It allows healthcare workers to make informed decisions quickly, even when advanced diagnostic tools are unavailable. However, the retrospective nature of the study, the inclusion of only hospitalized patients from one center, and the paucity of laboratory parameter data like the neutrophil-to-lymphocyte ratio in medical records were major limiting factors. Future studies with broader patient populations are needed to confirm these findings and establish more definitive conclusions.
